# Measuring students' perceptions of virtual reality for learning anatomy using the general extended technology acceptance model for E‐learning

**DOI:** 10.1002/ase.70045

**Published:** 2025-05-23

**Authors:** Sarah Alturkustani, Ashley Durfee, Olivia F. O'Leary, Siobhain M. O'Mahony, Conor O'Mahony, Mutahira Lone, Andreea Factor

**Affiliations:** ^1^ Department of Anatomy and Neuroscience University College Cork Cork Ireland; ^2^ APC Microbiome Ireland University College Cork Cork Ireland

**Keywords:** anatomy learning, E‐learning, GETAMEL, perception, virtual reality

## Abstract

Anatomy is fundamental to medical disciplines. However, its complexity can be challenging to learners, and traditional anatomy teaching may not be easily accessible. Virtual Reality has the potential to supplement anatomy education, but its effectiveness depends on students' willingness to accept it. This study aimed to measure students' perceptions of using anatomy software, 3D Organon, for learning anatomy, the factors influencing their acceptance of 3D Organon, and their perceived improvement in understanding eye and ear anatomy. Data were collected from eight MSc in Human Anatomy and nine BSc in Neuroscience students who used 3D Organon to learn eye and ear anatomy. 3D Organon acceptance was assessed using descriptive statistics for the variables of the General Extended Technology Acceptance Model for E‐Learning: perceived ease of use, perceived usefulness, behavioral intention, self‐efficacy, computer anxiety, and enjoyment. Correlation and regression analyses of the GETAMEL data determined factors predicting acceptance. Results showed that students highly accepted 3D Organon, with enjoyment being the highest rated (*μ* = 1.9). Self‐efficacy, enjoyment, and computer anxiety accounted for 91% of perceived ease of use, with enjoyment being the best predictor (*β* = 0.78). Self‐efficacy, enjoyment, and perceived ease of use significantly influenced perceived usefulness, with perceived ease of use being the strongest predictor (*β* = 1.02). Both perceived ease of use and perceived usefulness contributed to 85% of behavioral intention to use 3D Organon, with perceived ease of use being the stronger predictor (*β* = 1.01). These findings suggest that students highly accepted 3D Organon for learning anatomy, with enjoyment and ease of use being essential factors influencing their willingness to use it.

## INTRODUCTION

### Modern teaching approaches in anatomy education

Traditionally, anatomy education has relied on cadaveric‐based teaching, such as dissections and prosections.^1^ These teaching methods are well‐researched within the field, and their importance in medical education is well documented.^1^, ^2^, ^3^, ^4^, ^5^ Didactic lectures and textbooks are also widely used^6^ and are essential for learning anatomy.^1^, ^7^ However, the sole use of these modalities in the modern anatomy curriculum is being reconsidered, particularly in light of technological knowledge and expectations of younger generations.^3^, ^8^, ^9^ Moreover, using cadaver‐based teaching is significantly limited due to financial, ethical, and supervisory challenges.^5^, ^10^ While students can effectively learn anatomy using traditional modalities,^3^, ^4^, ^7^, ^11^ complex anatomical structures like the head and neck structures may be more effectively observed using advanced visualization technology.^11^, ^12^ The emergence of technology in education has changed how students approach anatomy learning, and its successful implementation is already evident in the literature.^1^, ^9^, ^12^, ^13^, ^14^, ^15^, ^16^, ^17^, ^18^ Technology‐enhanced learning employs video tutorials, e‐learning, and digital anatomy models such as computer‐based models, Virtual Reality, Augmented Reality, and Mixed Reality.^10^, ^19^, ^20^, ^21^, ^22^, ^23^

### Virtual Reality technology in anatomy education

Three‐ Dimensional Visualization Technology (3DVT) fully immerses the user in a simulated environment resembling the real world.^10^ It assists the teachers in engaging with various cohorts of students^12^ and allows the users to experience and interact with a 3D virtual environment using a head‐mounted device.^12^, ^24^ It has been widely used for medical and surgical training^25^, ^26^, ^27^, ^28^, ^29^ and anatomy education,^23^, ^30^, ^31^, ^32^, ^33^ with positive learning results documented in both fields. VR technology is believed to facilitate the learning of complex anatomical structures (i.e., eye, ear, brain, heart, etc.) and their spatial relationships,^12^, ^17^, ^32^, ^34^, ^35^ and therefore, it can effectively complement dissection and prosections in learning complex anatomical structures.^11^, ^12^, ^36^ In addition, 3DVT allows the user to manipulate objects in a continuous visual transformation,^37^ contributing to the construction of a mental representation of anatomical structures.^37^, ^38^, ^39^

The ongoing discussions about the impact of VR‐based anatomy modalities indicate their emerging role in anatomy education. Therefore, it is imperative to evaluate students' acceptance of using VR for anatomy learning and examine the factors influencing it.

### Technology evaluation using Technology Acceptance Models

The Technology Acceptance Model (TAM) has been widely used to explain technology adoption and usage. The initially proposed model by Davis (1986) was based on the Theory of Reasoned Action,^40^, ^41^ which stated that a person's behavior‐related beliefs drove the actual behavior. Davis (1986) concluded that system usage was explained by the user's beliefs/perceptions about the system's usefulness and ease of use^40^. The association between the perceived ease of use and perceived usefulness of a system and the self‐reported system usage was further consolidated by Davis^42^, establishing the perceived ease of use and perceived usefulness as fundamental determinants of system usage and adoption.^42^

Perceived ease of use refers to the extent to which an individual considers using a particular system would be free from effort.^42^ Perceived usefulness refers to the degree to which an individual believes a specific system enhances their work performance.^42^ The influence of perceived ease of use on perceived usefulness is evident in the literature.^43^, ^44^, ^45^, ^46^, ^47^

**FIGURE 1:**
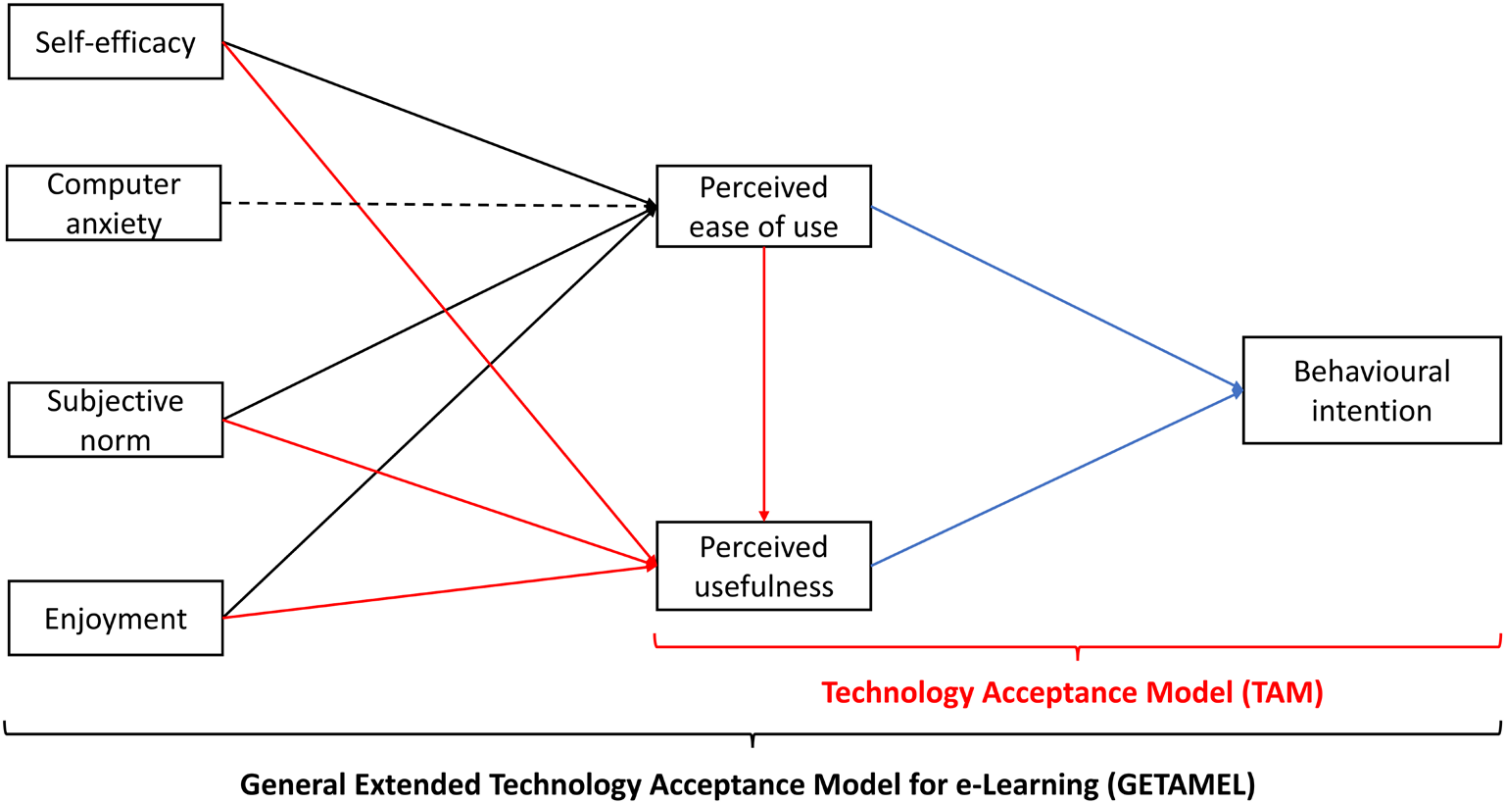
The General Extended Technology Acceptance Model for E‐Learning (GETAMEL) proposed by Abdullah and Ward^52^ based on the final Technology Acceptance Model (TAM) proposed by Venkatesh and Davis.^48^ Solid lines represent positive associations, and dashed lines represent negative associations.

Later, the behavioral intention, which indicates a person's willingness to complete a behavior, was proposed as an additional predictor of system use (Figure 1).^48^, ^49^ The association between perceived ease of use, perceived usefulness, and behavioral intention has been well documented.^45^, ^46^, ^47^, ^50^, ^51^

The TAM postulates that a system perceived as useful for enhancing job performance and perceived as easy to use is more likely to be accepted by users.^42^ Further work by Davis and colleagues has identified external factors such as system features and previous experience as potential predictors of perceived ease of use and perceived usefulness.^48^, ^53^

Understanding the factors influencing people to accept and use an information system or a technology is crucial;^54^ therefore, many researchers have extended TAM to include various external variables.^55^, ^56^ In their systematic review,^52^ selected five factors as the most commonly used external variables demonstrating significant associations with perceived ease of use and perceived usefulness: self‐efficacy, computer anxiety, subjective norm, enjoyment, and experience, and used these variables to propose the General Extended Technology Acceptance Model for e‐Learning (GETAMEL)^52^ (Figure 1). Using these five external factors, GETAMEL was validated in a subsequent study in the context of e‐portfolios and was supported as a robust and reliable model for explaining variability in users' acceptance and behavioral intention to use e‐portfolios.^55^

Self‐efficacy denotes the beliefs a person has about their capabilities to perform a particular behavior or task successfully,^57^ and its effects on perceived ease of use and perceived usefulness of various e‐learning systems are well supported by evidence.^58^, ^59^, ^60^, ^61^, ^62^, ^63^, ^64^, ^65^, ^66^, ^67^, ^68^

Computer anxiety refers to a person's apprehension about using computers.^69^ While the negative effect of computer anxiety on perceived ease of use is well documented,^70^, ^71^, ^72^, ^73^, ^74^ its effect on perceived usefulness in e‐learning is not well supported.^52^ Therefore, the association between computer anxiety and perceived usefulness was excluded from the GETAMEL and, thus, from the present study.

Subjective norm refers to the degree to which an individual perceives social pressure in performing a behavior.^70^ Therefore, a student's tendency to use or not use an e‐learning system can be influenced by the opinions of people in the student's environment.^55^, ^75^ Previous research across various e‐learning systems has demonstrated its impact on perceived ease of use and perceived usefulness.^68^, ^76^, ^77^, ^78^, ^79^

Enjoyment refers to the degree to which an activity using a specific technology is perceived to be enjoyable.^80^ Research in technology‐enhanced learning employing the TAM has consistently demonstrated the significant impact of enjoyment on perceived ease of use and perceived usefulness.^58^, ^80^, ^81^, ^82^, ^83^, ^84^, ^85^, ^86^

Technology experience is determined by an individual's duration and familiarity with a specific technology.^87^ Technology experience has been shown to moderate e‐system adoption^88^, ^89^ and thus was included in the TAM. Although GETAMEL considered the influence of experience on students' perception of e‐learning, its impact on perceived ease of use was only significant.^55^

In addition to the primary relationships within the original TAM, GETAMEL postulates a positive correlation between self‐efficacy, enjoyment, subjective norm, and experience and each perceived ease of use and perceived usefulness, as well as a negative correlation between computer anxiety and perceived ease of use^52^, ^55^ (Figure 1).

### Study objectives

Understanding the complexity of small anatomical structures, such as the eye and ear, and visualizing their intricate anatomical relationships can be achieved more easily with virtual anatomy models than traditional learning methods. However, for VR anatomy software to be successfully integrated into anatomy education, it is essential to assess students' acceptance of this learning tool. This study aimed to evaluate students' perceptions of and acceptance toward using VR anatomy software (3D Organon Version 2023.1.1.2, developed by Medis Media Pty Ltd) for learning eye and ear anatomy. The research sought to compare the perceptions between two student cohorts, identify the factors influencing their perceptions and acceptance of the software, and examine its impact on their self‐perceived comprehension of eye and ear anatomy.

To achieve these objectives, the study applied the GETAMEL framework proposed by Abdullah and Ward^52^ to investigate the factors influencing students' perceptions, acceptance, and future use of 3D Organon.

### Research questions

The following research questions were developed to achieve the objectives:

1. How do students perceive the anatomy software 3D Organon as an educational resource for learning anatomy, and how do these perceptions vary across disciplines?
2. Which factors influence the acceptance and intention to use 3D Organon for anatomy learning?
3. Does learning the eye and ear anatomy with 3D Organon improve students' self‐perceived anatomy comprehension of these structures?

### Research model

**FIGURE 2:**
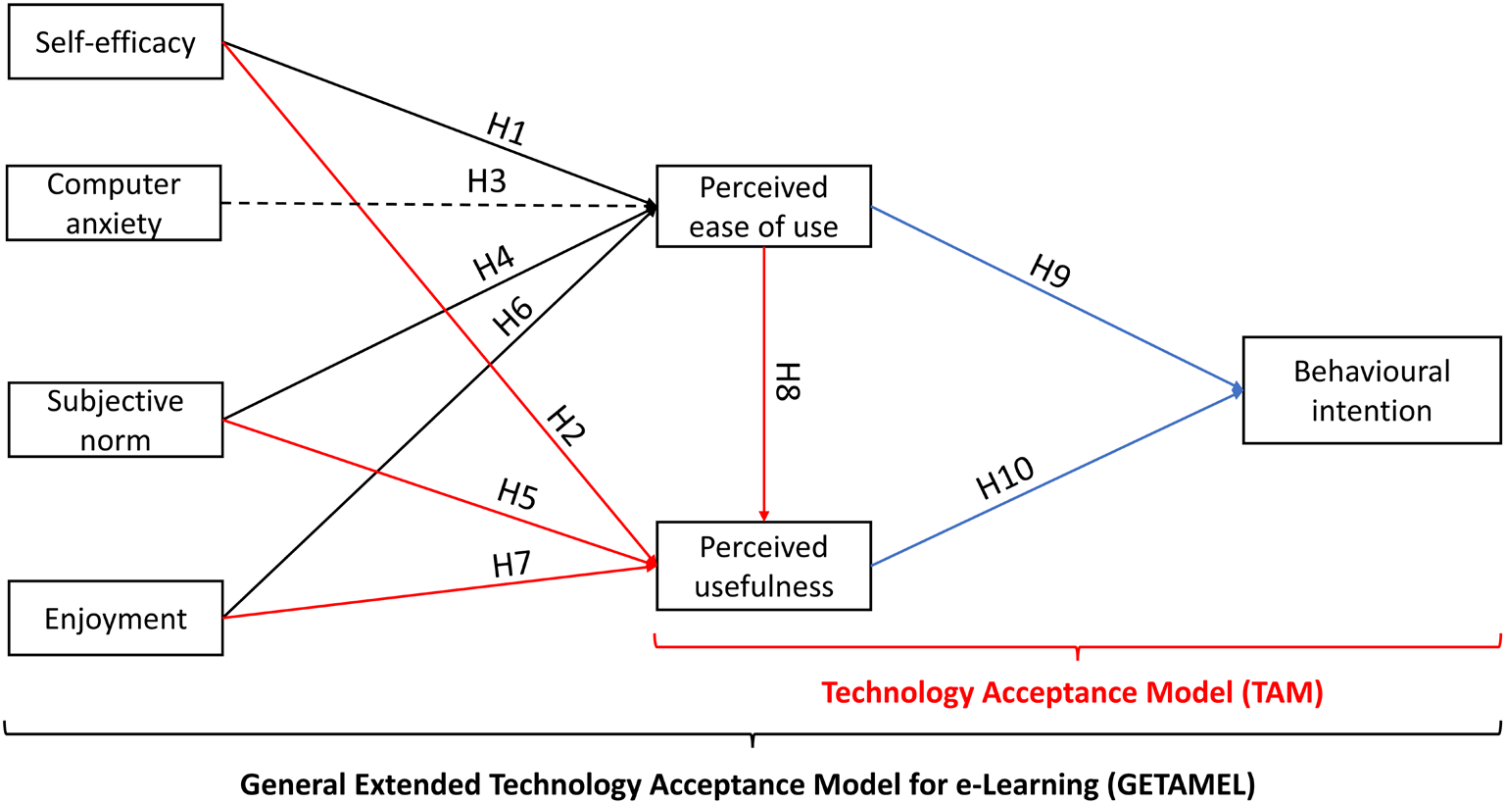
A summary of the research model and hypotheses. The solid lines represent positive associations, and the dashed lines represent negative associations. Black arrows represent predictors of perceived ease of use, red arrows represent predictors of perceived usefulness, and blue arrows represent predictors of behavioral intention to use VR.

The present study considers the relationships the TAM and GETAMEL models address to achieve study objectives. Self‐efficacy, computer anxiety, subjective norm, and enjoyment from the GETAMEL were considered external factors; however, experience was excluded due to the novelty of VR in anatomy learning. Figure 2 presents a summary of the research model and hypotheses.

The association between self‐efficacy and each perceived ease of use and perceived usefulness in e‐learning indicates that the higher the students' confidence in operating an e‐learning system, the more likely they are to perceive it as easy and useful. Therefore, we hypothesize (H):

Self‐efficacy positively influences perceived ease of use of 3D Organon.

Self‐efficacy positively influences perceived usefulness of 3D Organon.

Considering the context of e‐learning, the less anxious the user is about using an e‐learning system, the more they perceive it as easy to use; we hypothesize:

Computer anxiety negatively influences perceived ease of use of 3D Organon.

As a student's tendency to engage or not with an e‐learning system is influenced by the opinions of people in the student's environment, we hypothesize:

Subjective norm positively influences perceived ease of use of 3D Organon.

Subjective norm positively influences perceived usefulness of 3D Organon.

An enjoyable system is more likely to be perceived as easy and useful. Therefore, we hypothesize:

Enjoyment positively influences perceived ease of use of 3D Organon.

Enjoyment positively influences perceived usefulness of 3D Organon.

Considering TAM in the e‐learning context, an e‐learning system perceived as simple and useful is more likely to be accepted and used for learning. Thus, we hypothesize:

Perceived ease of use positively influences perceived usefulness of 3D Organon.

Perceived ease of use positively influences behavioral intention to use 3D Organon.

Perceived usefulness positively influences behavioral intention to use 3D Organon.

## RESEARCH METHODS

### Participants

MSc in Human Anatomy and third‐year BSc Neuroscience students were recruited during the 2022–2023 academic year at University College Cork, Ireland. An invitation email was sent to both cohorts. The MSc students were enrolled in the Human Gross Anatomy II module, while the Neuroscience students were in the Human Regional Neuroanatomy module. Both modules involved didactic lectures and anatomy laboratory sessions. A typical anatomy laboratory session included bone specimens, prosected specimens, and computer‐assisted learning with McGraw Hill Connect Anatomy & Physiology Revealed (v4.0 Online HTML VERSION). However, plastic models were mainly used for eye and ear anatomy. Medical demonstrators guided the students in exploring the anatomical structures and innervation of the eye and ear, followed by independent study of the models. No exclusion criteria were applied, and ethical approval was obtained from the Social Research Ethics Committee at University College Cork (Log 2023‐076). All students provided verbal and written consent.

### VR sessions and data collection

**FIGURE 3:**
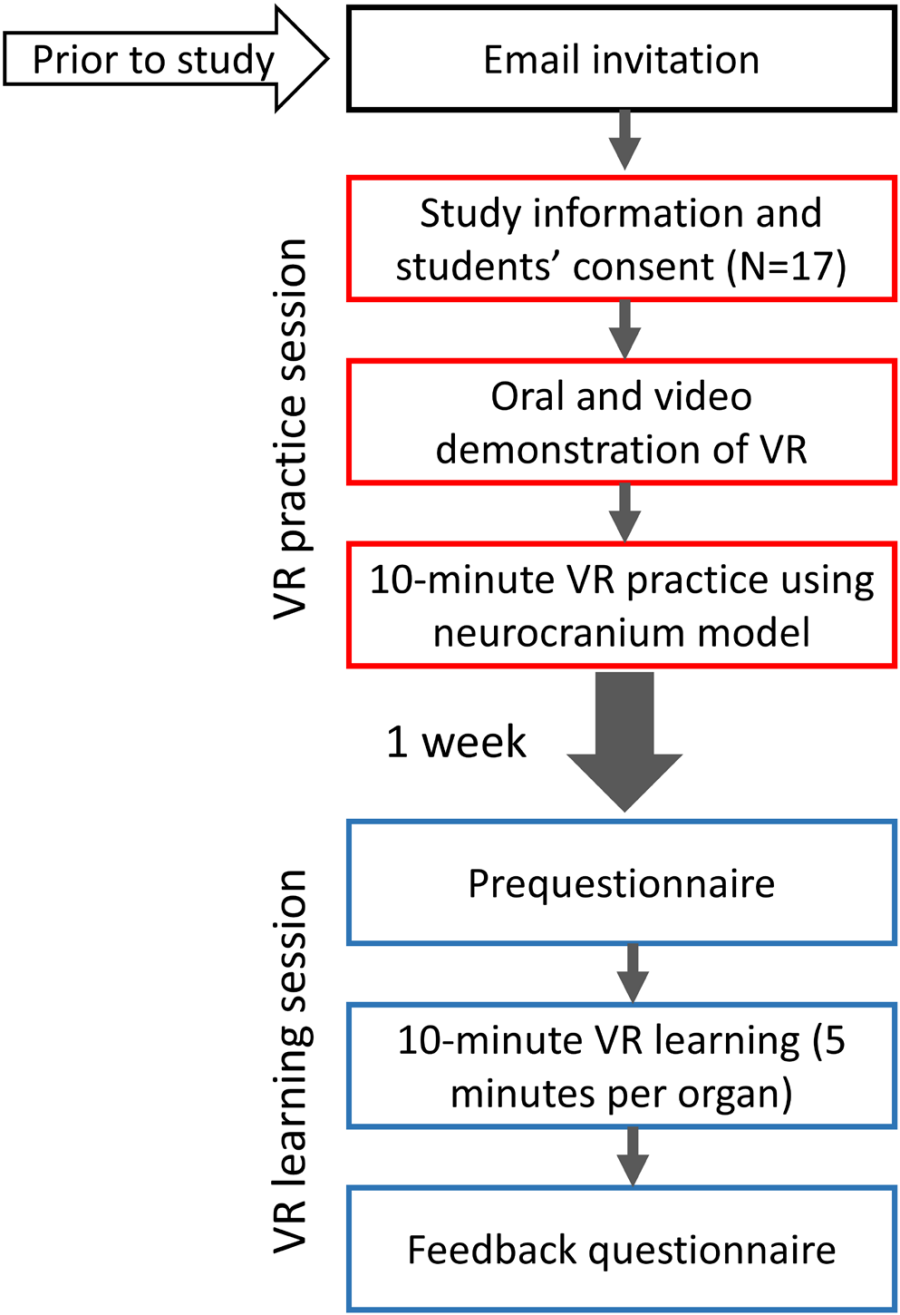
A schematic overview of the study. The red boxes represent the practice session, and the blue boxes represent the learning session. The sessions were one week apart.

**FIGURE 4:**
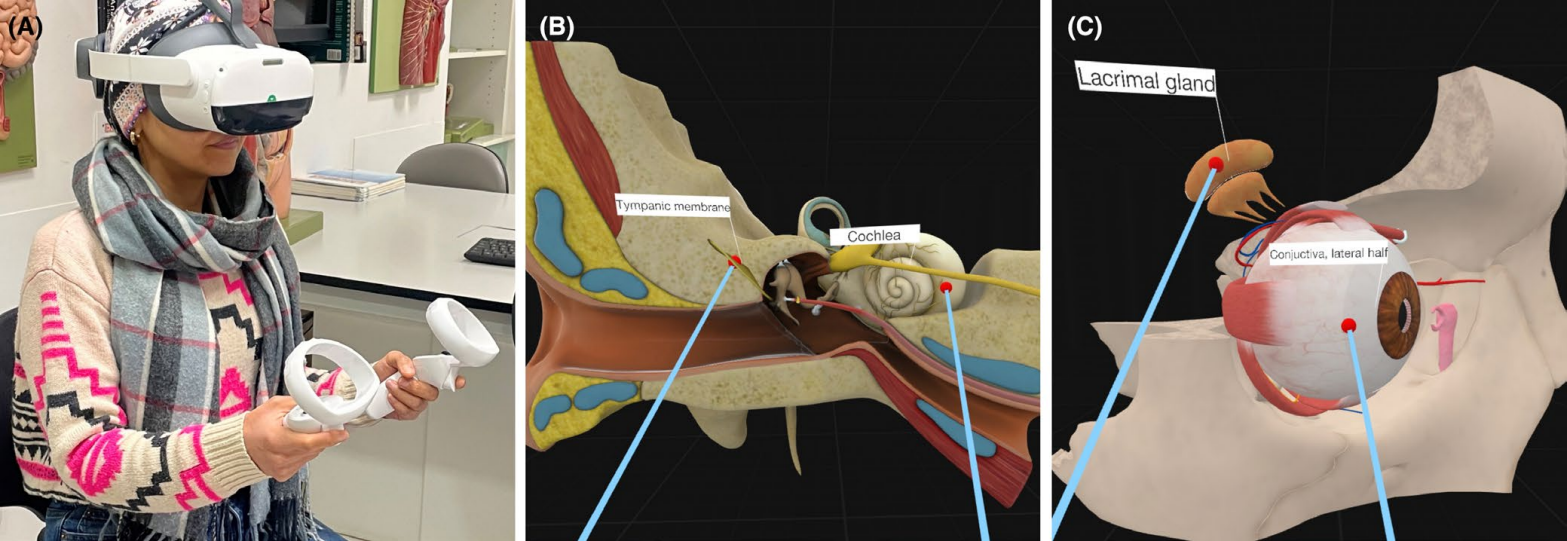
(A) VR station: The VR device and 3D anatomy software (3D Organon) were used for the practice and learning sessions. (B and C) Screenshots of the 3D Organon software showing visualization and virtual dissection of the eye and ear anatomy performed during the learning session.

Each student cohort participated in two anatomy laboratory sessions held one week apart, each including a VR session (Figure 3). The first anatomy laboratory session focused on skull anatomy and included a VR practice session. Initially, the students watched a video demonstration on using the anatomy software, 3D Organon. Two VR stations were available, each equipped with one PICO New Generation 3 Virtual Reality headset (developed by Pico Technology Co. Ltd.) (Figure 4A). A research team member was present at each station to provide brief instructions on operating the VR headset and the anatomy software, 3D Organon. Students rotated through the VR stations, each allocated 10 min to explore, identify, and virtually dissect the skull and facial muscles. While two students used the VR system at a time, the others engaged in computer‐assisted learning with McGraw Hill Connect Anatomy & Physiology Revealed to study the same anatomical structures.

The second anatomy laboratory session (VR learning session) was held a week later and followed a similar format, focusing on eye and ear anatomy. Before attending the VR sessions, the students completed a pre‐questionnaire, which is detailed in a later section. Each student was allocated 10 min (5 min per organ) to identify and virtually dissect eye and ear structures (Figure 4B,C). Similar to the first laboratory session, students not using the VR system studied the same content using McGraw Hill Connect Anatomy & Physiology Revealed. After the session, each student completed a feedback questionnaire, described in a later section (Figure 3 shows a schematic overview of the study).

### Instrumentation

Two questionnaires were used for the current study. The pre‐questionnaire was administered before the VR learning session and consisted of 2 sections. Section 1 consisted of 20 questions and collected data on student demographics, previous anatomy experience, use of 3D anatomy atlases, medical history of learning disabilities, loss of balance or visual conditions, and VR experience and associated physical discomfort. Section 2 measured students' self‐perceived eye and ear anatomy comprehension on a 3‐point scale, where 1 = not understood, 2 = partially understood, and 3 = well understood. Students were asked to rate their comprehension of specific eye structures, including the extraocular muscles, fibrous layer, vascular layer, nervous layer, lens, and vitreous body. Similarly, they rated their comprehension of ear structures, including the tympanic membrane, tympanic cavity, auditory ossicles, inner ear, cochlea, vestibule, and semi‐circular canals. The pre‐questionnaire is provided as supplementary material.

The feedback questionnaire was administered immediately following the VR learning session and consisted of 2 sections. Section 1 comprised 7‐point Likert scale items ranging from 1 = strongly agree to 7 = strongly disagree to measure the external variables outlined in the General Extended Technology Acceptance Model of E‐Learning (excluding experience) and the main variables outlined in the Technology Acceptance Model, totaling seven variables with 22 underlying items (Table 2). This section also included six open‐ended questions designed to collect additional insights into students' experiences with 3D Organon and VR. These questions aimed to explore the following aspects: students' opinions on 3D Organon, including likes and dislikes, the anatomy structures students identified as most suitable for VR‐based learning, their comparison of VR with traditional teaching methods, the extent to which VR and 3D Organon motivated them to learn anatomy, and suggestions for improvement. Section 2 of the feedback questionnaire measured students' self‐perceived eye and ear anatomy comprehension using the same scale and items as the pre‐questionnaire. The feedback questionnaire is provided as supplementary material.

Two research members developed the instruments, and three institutional faculty members with anatomy teaching experience reviewed them for face validity. Adjustments were made where appropriate based on the feedback provided.

### Data analysis

Descriptive statistics of student demographics, self‐perceived anatomy comprehension ratings (measured on a 3‐point scale), and ratings on the 22 GETAMEL items (measured on a 7‐point scale) are presented using the mean and standard deviation (SD). Mean ratings of the self‐perceived eye and ear anatomy comprehension items were combined into a single composite score representing students' overall comprehension of the corresponding structure, with scores of 1–1.66 representing poor understanding, 1.67–2.33 representing partial understanding, and 2.34–3 representing full understanding of the eye and ear anatomy. These scores were used as dependent variables to compare students' pre‐ and post‐VR eye and ear anatomy comprehension. A composite score for each of the 7 GETAMEL variables was also calculated using the mean ratings of their corresponding items. These scores were used to measure students' perceptions of 3D Organon, where 1–1.85 represent strongly agree, 1.86–2.71 represent moderately agree, 2.72–3.57 represent slightly agree, 3.58–4.43 represent neutral, 4.44–5.29 represent slightly disagree, 5.3–6.15 represent moderately disagree, and 6.16–7 represent strongly disagree.

To explore cohort‐based differences in perceptions, the mean scores of the GETAMEL variables were used as dependent variables and compared using independent samples *t*‐tests. The association between the variables was first examined using scatterplots and correlation analysis to identify which external variables acted as predictors of perceived ease of use, perceived usefulness, and behavioral intention to use 3D Organon. Pearson correlation coefficients were interpreted based on Mukaka^90^. Where significant correlations were detected, simple linear regression analyses were conducted to ascertain the associations and determine the significance of predictors. Both student cohorts' GETAMEL data were pooled together for correlation and regression analyses. The normality of data was confirmed visually using histograms and normal Q‐Q plots. Therefore, parametric tests were used for analyses. All statistical analyses were performed at a level of significance *α* = 0.05 and were conducted using the Statistical Package for Social Scientists (SPSS), version 28.0 (190) (IBM Corp., Armonk, NY). The internal consistency of each GETAMEL variable was measured by Cronbach's alpha.

## RESULTS

### Demographics

Seventeen students (9 Neuroscience and 8 MSc) participated in the study. The median age of the neuroscience cohort was 21 (IQR = 1); 22.2% of them were female, and all were undergraduate students. The median age of the MSc cohort was 24 (IQR = 7); 62.6% of them were female, and most (87.5%) had a Bachelor's degree only. Both cohorts were familiar with eye and ear anatomy, and the majority (88.2%) reported minimal use of 3D anatomy atlases for studying. The majority (77.8%) of the BSc Neuroscience student cohort reported a previous experience with VR, whereas none of the MSc students had used VR before. Table 1 shows more detailed demographics.

**TABLE 1:** Demographic information collected using section 1 of the pre‐questionnaire.

Characteristics	MSc (*N* = 8)	Neuroscience (*N* = 9)	Total (*N* = 17)
Gender			
Male	37.5%	66.7%	52.9%
Female	62.5%	22.2%	41.2%
Not specified	0%	11.1%	5.9%
Country of origin			
Irish	62.5%	77.8%	70.6%
Non‐Irish	37.5%	22.2%	29.4%
Educational level			
2nd level qualification or equivalent	0%	100%	52.9%
Bachelor's degree	87.5%	0%	41.2%
Postgraduate degree	12.5%	0%	5.9%
Previous experience with eye and ear anatomy			
Yes	100%	100%	100%
No	0%	0%	0%
Frequency of digital 3D atlas use			
Occasionally	25%	0%	11.8%
Rarely	37.5%	88.9%	64.7%
Never	37.5%	11.1%	23.5%
Previous VR experience			
Yes	0%	77.8%	41.2%
No	100%	22.2%	58.8%
Previous history of learning disabilities, vision problems, or loss of balance			
Yes	50%	0%	23.5%
No	50%	100%	76.47%
VR‐related symptoms with previous VR use			
Yes	0%	11.11%	5.9%
No	0%	66.67%	35.3%
Not applicable^a^	100%	22.22%	58.8%

^a^ These students did not have a previous VR experience.

### Students' acceptance of 3D Organon

**FIGURE 5:**
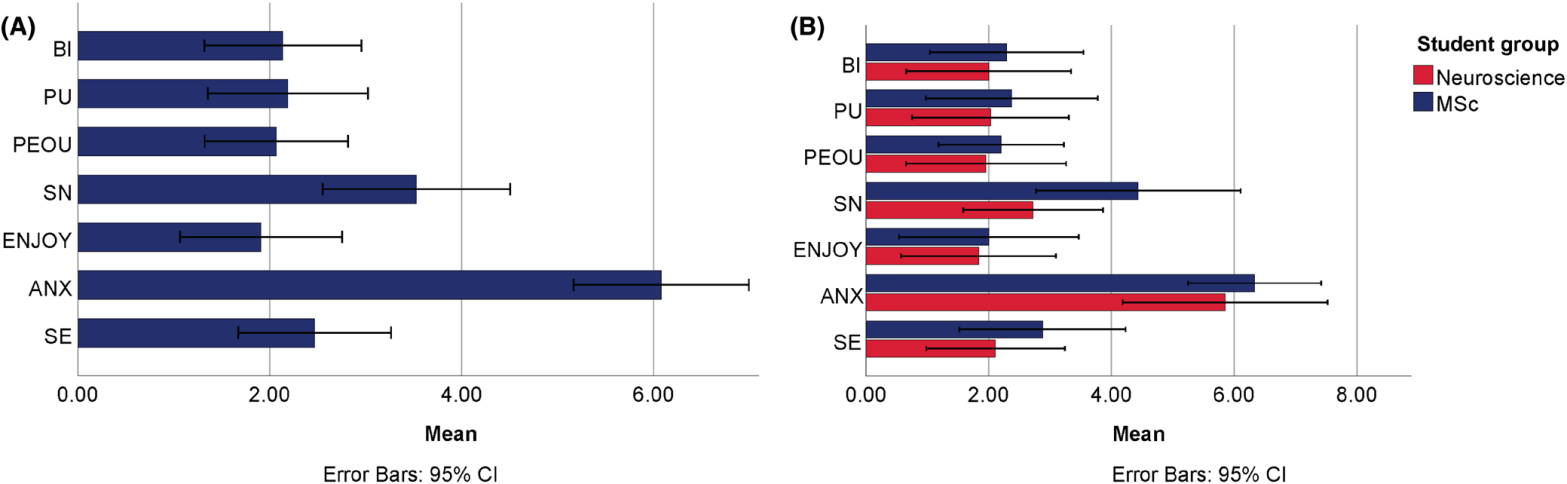
(A) Students' perceptions of 3D Organon based on the 7 GETAMEL variables. Bars represent the composite score of the variables calculated using the mean rating on its corresponding items. Mean scores 1–1.85 represent strongly agree, 1.86–2.71 represent moderately agree, 2.72–3.57 represent slightly agree, 3.58–4.43 represent neutral, 4.44–5.29 represent slightly disagree, 5.3–6.15 represent moderately disagree, and 6.16–7 represent strongly disagree. (B) Cohort‐based comparisons of 3D Organon perception in each of the 7 GETAMEL variables. No significant differences were found between the cohorts in the GETAMEL variables. ANX, computer anxiety; BI, behavioral intention; ENJOY, enjoyment; PEOU, perceived ease of use; PU, perceived usefulness; SE, self‐efficacy; SN, subjective norm. Error bars represent 95% confidence intervals (CI).

Table 2 shows the results of the GETAMEL items. The mean (±SD) for each of the 22 items and Cronbach's alpha for each of the 7 GETAMEL variables are presented. The highest level of agreement was reported on the following items: “working with VR anatomy software does not make me nervous,” *μ* = 1.76 (±1.6), “I find using the VR anatomy software enjoyable,” *μ* = 1.76 (±1.68), “assuming I had access to the VR anatomy software, I would use it for learning other anatomical topics,” *μ* = 1.88 (±1.54), and “Given that I had access to the VR anatomy software, I predict that I would use it for learning other anatomical topics,” *μ* = 1.88 (±1.54). Mean ratings and SD for the GETAMEL variables are presented in Figure 5A. Students reported agreement toward self‐efficacy, enjoyment, subjective norm, perceived ease of use, perceived usefulness, and behavioral intention to use 3D Organon, and disagreement toward computer anxiety, indicating positive perception of 3D Organon with enjoyment having the highest agreement, *μ* = 1.9 (±1.64) (Figure 5A). All variables had an acceptable level of internal consistency >0.8.^91^ Variable scores within each cohort are shown in Figure 5B. Comparisons between cohorts' perceptions of 3D Organon using independent samples *t*‐test showed no significant differences in any of the variables (*p* > 0.05) (Figure 5B).

**TABLE 2:** Students' perceptions of the 3D Organon collected using section 1 (the GETAMEL) of the feedback questionnaire.

Variables	Cronbach's alpha	Items	Mean	SD
Self‐efficacy	0.81	I am confident to use the VR anatomy software if there is no one around to show me how to do it	2.24	1.43
		I am confident to use the VR anatomy software even if I have never used such a system before	2.35	1.61
		I am confident to use the VR anatomy software even if I have only the software manual for reference	2.82	2.29
Computer anxiety	0.96	Working with VR anatomy software does not make me nervous^a^, ^b^	1.76	1.60
		VR anatomy software makes me uncomfortable	6.00	1.96
		VR anatomy software makes me uneasy	6.00	1.96
Enjoyment	0.98	I find using the VR anatomy software enjoyable^a^	1.76	1.67
		The actual process of using the VR anatomy software is pleasant	2.06	1.63
Subjective norm	0.91	People who influence my behavior would think that I should use the VR anatomy software	3.47	1.84
		I am influenced by my peers and my lecturers in using the VR anatomy software	3.59	2.12
Perceived ease of use	0.93	I find it easy to use the VR headsets to navigate through the anatomy software	2.12	1.49
		My interaction with the VR anatomy software is clear and understandable	2.12	1.76
		I would find the VR anatomy software to be flexible to interact with	2.12	1.45
		It would be easy for me to become skillful at using the VR anatomy software	2.00	1.76
		I would find the VR anatomy software helpful in visualizing the structures	2.00	1.76
Perceived usefulness	0.98	Using the VR anatomy software allows me to accomplish learning tasks more quickly	2.35	1.90
		Using the VR anatomy software improves my learning performance	2.18	1.59
		Using the VR anatomy software enhances my effectiveness in learning	2.12	1.53
		Using the VR anatomy software would make learning easier	2.12	1.65
Behavioral intention	0.94	Assuming I had access to the VR anatomy software, I would use it for learning other anatomical topics^a^	1.88	1.53
		Given that I had access to the VR anatomy software, I predict that I would use it for learning other anatomical topics^a^	1.88	1.53
		I plan to use the VR anatomy software in the future	2.65	1.93

*Note*: The GETAMEL items are rated on a 7‐point Likert scale: 1–1.85 represent strongly agree, 1.86–2.71 represent moderately agree, 2.72–3.57 represent slightly agree, 3.58–4.43 represent neutral, 4.44–5.29 represent slightly disagree, 5.3–6.15 represent moderately disagree, and 6.16–7 represent strongly disagree.

^a^ Items with the highest agreement.

^b^ These items have been reversed.

## FACTORS INFLUENCING THE PERCEPTION AND USE OF 3D ORGANON

### Correlations

The associations between the GETAMEL variables were assessed using Pearson's correlation (Table 3). Self‐efficacy was positively correlated with perceived ease of use and perceived usefulness, while computer anxiety showed negative correlations with these variables. Enjoyment was positively correlated with perceived ease of use and perceived usefulness. Additionally, perceived ease of use was strongly correlated with perceived usefulness and behavioral intention to use 3D Organon. Perceived usefulness also correlated positively with behavioral intention to use 3D Organon. No linear relationship was observed between subjective norm and either perceived ease of use or perceived usefulness, leading to its exclusion from further analyses.

**TABLE 3:** Estimated Pearson correlations between the GETAMEL variables.

Variables	Self‐efficacy	Computer anxiety	Enjoyment	Perceived ease of use	Perceived usefulness	Behavioral intention
Self‐efficacy	1	−0.606**	0.510*	0.751**	0.608**	0.557*
Computer anxiety		1	−0.876**	−0.885**	−0.807**	−0.886**
Enjoyment			1	0.878**	0.896**	0.947**
Perceived ease of use				1	0.915**	0.923**
Perceived usefulness					1	0.889**
Behavioral intention						1

** Correlation is significant at the 0.01 level (2‐tailed).

* Correlation is significant at the 0.05 level (2‐tailed).

These results indicate that students reporting high self‐efficacy and enjoyment scores also reported high perceived ease of use and perceived usefulness scores. Students reporting low computer anxiety scores also reported high perceived ease of use scores. Students reporting high perceived ease of use and perceived usefulness scores also reported high behavioral intention scores. In addition, moderate to high correlations were found between self‐efficacy, computer anxiety, and enjoyment. All correlations were either significant (*p* < 0.05) or highly significant (*p* < 0.01).

### Hypothesis testing

Simple linear regression analyses were conducted to test the hypotheses (H1–H10), with the results summarized in Table 4. Hypotheses H1, H3, and H6 were supported, as self‐efficacy, computer anxiety, and enjoyment were significant predictors of perceived ease of use. Similarly, H2, H7, and H8 were supported, as self‐efficacy, enjoyment, and perceived ease of use significantly predicted perceived usefulness.

**TABLE 4:** Results of the simple regression analyses for hypothesis testing.

Hypotheses		Unstandardized coefficients		Model summary					Conclusion
		*β*	*t*	*F*	*p* Value	*R*	*R* ^2^	*SE*	
H1	SE → PEOU	0.71**	4.401	19.366	<0.001	0.75	0.56	0.99	Hypothesis supported
H2	SE → PU	0.64*	2.969	8.813	0.01	0.61	0.37	1.3	Hypothesis supported
H3	ANX → PEOU	−0.72**	−7.361	54.183	<0.001	0.89	0.78	0.7	Hypothesis supported
H6	ENJOY → PEOU	0.78**	7.113	50.599	<0.001	0.88	0.77	0.72	Hypothesis supported
H7	ENJOY → PU	0.89**	7.829	61.3	<0.001	0.9	0.8	0.74	Hypothesis supported
H8	PEOU → PU	1.02**	8.766	76.851	<0.001	0.92	0.84	0.68	Hypothesis supported
H9	PEOU → BI	1.01**	9.277	86.054	<0.001	0.92	0.85	0.63	Hypothesis supported
H10	PU → BI	0.87**	7.539	56.837	<0.001	0.89	0.79	0.75	Hypothesis supported

*Note*: A summary of the hypothesis testing. All regression analyses were significant and supported the hypotheses. H4 and H5 were excluded as no linear relationship was detected. *β*, beta weight; *R*, correlation coefficient; *R* ^2^, coefficient of determination; *SE*, standard error.

Abbreviations: ANX, computer anxiety; BI, behavioral intention to use VR; ENJOY, enjoyment; PEOU, perceived ease of use; PU, perceived usefulness; SE, self‐efficacy.

* *p* < 0.05.

** *p* < 0.01.

H9 and H10 were also supported, with perceived ease of use and perceived usefulness significantly predicting behavioral intention to use 3D Organon. Enjoyment (*β* = 0.78) was the strongest predictor of perceived ease of use, while perceived ease of use (*β* = 1.02) emerged as the strongest predictor of both perceived usefulness and behavioral intention to use 3D Organon (*β* = 1.01). H4 and H5 were rejected due to the absence of a linear relationship between subjective norm and both perceived ease of use and perceived usefulness.

### Final model

**FIGURE 6:**
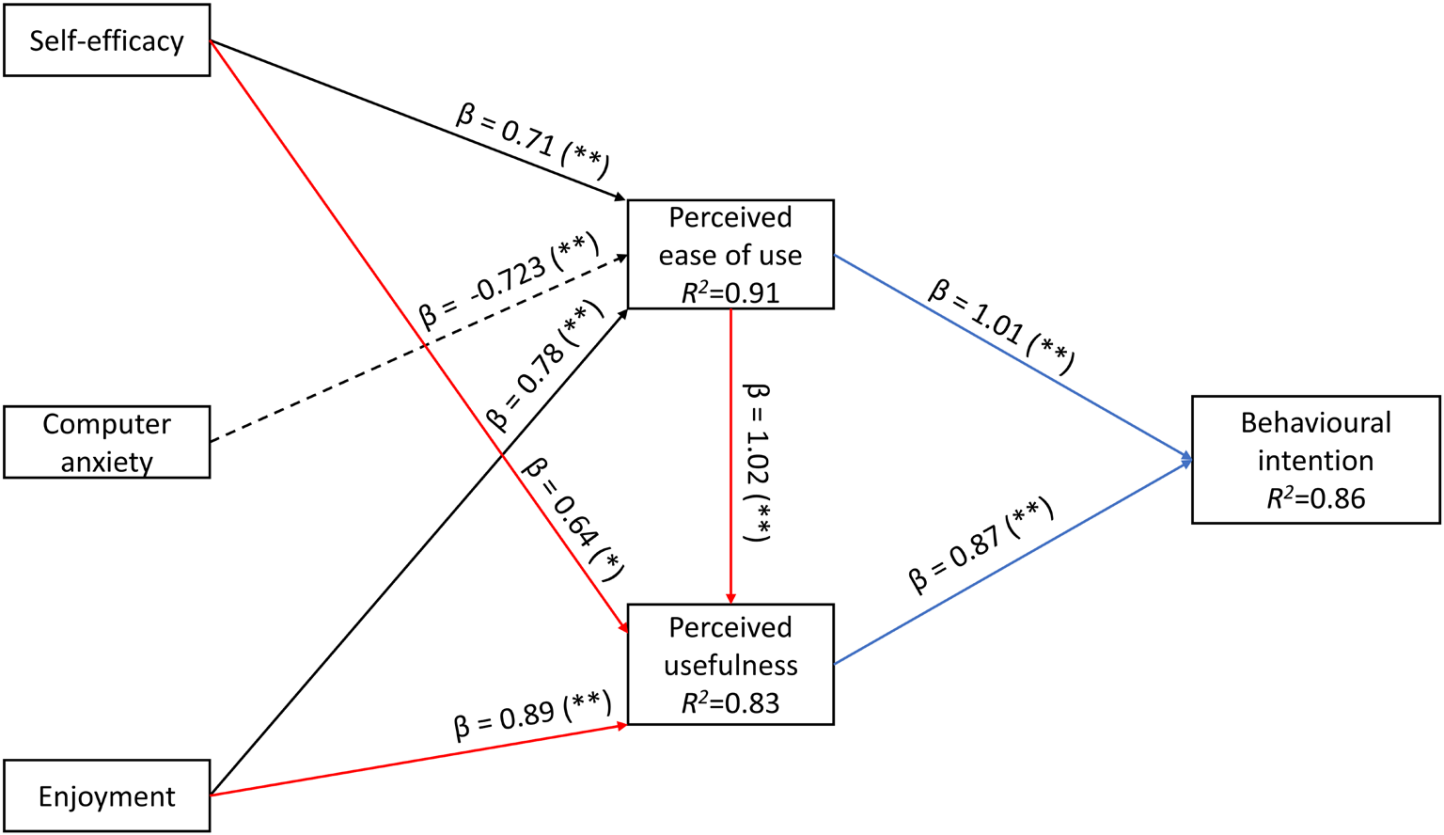
The final research model based on simple regression analyses. The solid lines represent positive associations, and the dashed lines represent negative associations. Black arrows represent the predictors of perceived ease of use, red arrows represent predictors of perceived usefulness, and blue arrows represent predictors of the behavioral intention to use 3D Organon. *p* Values are presented in the parentheses (*p < 0.05; **p < 0.01). *R* ^2^ (coefficient of determination) shows the percentage of the variance explained by the predictors. *β*, beta coefficient.

Three multiple regression models were performed to examine how various predictors collectively contributed to the variances in perceived ease of use, perceived usefulness, and behavioral intention to use 3D Organon. Figure 6 summarizes the final model.

In the first regression model, self‐efficacy, computer anxiety, and enjoyment collectively explained 91% of the variance in perceived ease of use (*R* ^2^ = 0.91, *F*_3_ = 42.9, *p* < 0.001). The largest Variance Inflation Factor (VIF) in this model was 5, indicating no significant multicollinearity issues.^92^

In the second regression model, perceived ease of use was excluded due to its high VIF of 9.51, indicating multicollinearity with other predictors. Therefore, this model focused on self‐efficacy and enjoyment as predictors of perceived usefulness. Together, they accounted for 83% of the variance in perceived usefulness (*R* ^2^ = 0.83, *F*_2_ = 35.25, *p* < 0.001). The VIF values for self‐efficacy (1.3) and enjoyment (1.3) were within acceptable limits.^92^

The third regression model examined the behavioral intention to use 3D Organon, which was explained by perceived ease of use and perceived usefulness, accounting for 86% of the variance (*R* ^2^ = 0.86, *F*_2_ = 44.54, *p* < 0.001). The VIF values for perceived ease of use (6.12) and perceived usefulness (6.12) were within the acceptable range.^92^

The remaining variances in each of the dependent variables—perceived ease of use (9%), perceived usefulness (17%), and behavioral intention to use 3D Organon (14%)—may be attributed to unmeasured or unknown factors.

### Anatomy comprehension

Table 5 shows the two student cohorts' mean self‐perceived eye and ear anatomy comprehension scores. The improvement in anatomy comprehension was analyzed using a two‐tailed paired samples *t*‐test. Overall, the mean pre‐ and post‐VR eye comprehension scores were 2.38 (±0.53) and 2.7 (±0.39), respectively. The mean scores for pre‐ and post‐VR ear comprehension were 2.32 (±0.53) and 2.76 (±0.4), respectively. Analysis of pooled data shows significant improvements in self‐perceived anatomy comprehension (*p* < 0.05) with a medium effect size (0.54) and (0.62) for the eye and ear, respectively. However, the cohort‐based analysis showed that only the MSc cohort reported significant improvements in self‐perceived ear comprehension (*p* = 0.014).

**TABLE 5:** Comparisons of self‐perceived comprehension scores before and after the VR learning session.

Cohort (*N*)	Structure	Mean (±SD) pre‐VR comprehension	Mean (±SD) post‐VR comprehension	Results
Neuroscience (9)	Eye	2.41 (±0.36)	2.65 (±0.48)	*t*_8_ = −1.579, *p* = 0.153, CI −0.59 to 0.11
	Ear	2.51 (±0.34)	2.65 (±0.49)	*t*_8_ = −1.029, *p* = 0.334, CI −0.46 to 0.18
MSc (8)	Eye	2.35 (±0.69)	2.85 (±0.24)	*t*_7_ = −2.323, *p* = 0.053, CI −1.03 to 0.009
	Ear	2.11 (±0.65)	2.88 (±0.26)	*t*_7_ = −3.242, *p* = 0.014, CI −1.34 to −0.21
Total (17)	Eye	2.38 (±0.53)	2.75 (±0.39)	*t*_16_ = −2.802, *p* = 0.013, CI −0.64 to −0.09
	Ear	2.32 (±0.53)	2.76 (±0.4)	*t*_16_ = −2.898, *p* = 0.01, CI −0.76 to −0.12

*Note*: Self‐perceived anatomy comprehension was measured on a scale from 1 to 3; 1–1.66 represent poor understanding, 1.67–2.33 represent partial understanding, and 2.34–3 represent full understanding of the eye and ear anatomy.

Abbreviations: CI, 95% confidence interval; *N*, sample size; SD, standard deviation.

### Responses to open‐ended questions

Most students' responses indicated a positive VR learning experience and a motivation to learn using 3D Organon. Compared with traditional methods, many students viewed 3D Organon as more effective for its unique features, while others saw the value of 3D Organon as a supplementary resource. Some responses indicated physical discomfort experienced during the VR session, while others highlighted potential improvements to the VR system. Table 6 shows students' quotes obtained from their responses to the open‐ended questions.

**TABLE 6:** Examples of student responses to the open‐ended questions from the feedback questionnaire.

Questions	Student response examples	Cohort
1. Which aspect of the VR anatomy software did you like the most and why?	“I liked being able to zoom in and visualize structures that are difficult to view with the naked eye.”	MSc of Anatomy
2. Which aspect of the VR anatomy software did you like the least and why?	“The slight eye strain.”	BSc of Neuroscience
3. What anatomical topic/structure do you think the VR would be an effective learning tool for and why?	“Structures that we don't often see inside, I think it would help more w internal structures of organs and tissues than overall anatomical structure.”	MSc of Anatomy
4. How does VR compare to conventional methods in learning the eye and ear anatomy? (i.e., lectures, plastic models, textbooks)	“I think it's better than the standard methods, it's a lot more interactive and allows you to take your time understanding it.”	BSc of Neuroscience
5. Using VR, do you feel more motivated to learn anatomy?	“Somewhat, if readily available, I would work that into my study plan.”	MSc of Anatomy
6. How can we improve your anatomy learning experience using VR?	“Try to fix the glitches. Didn't occur too much but it was hard to find the structures after they glitched out. Overall was a good experience.”	BSc of Neuroscience

## DISCUSSION

The current study aimed to measure students' perception and acceptance of using 3D Organon to learn anatomy, identify the factors influencing 3D Organon acceptance and perception, and evaluate students' self‐perceived comprehension of eye and ear anatomy after learning with 3D Organon. Assessing both the acceptance of technological learning resources and the factors influencing acceptance is significant because the effectiveness of these tools relies on their usage. The current study used the GETAMEL to examine 3D Organon acceptance and perception and measure students' behavioral intention to use it for future learning. Variables outlined in the GETAMEL, including self‐efficacy, computer anxiety, enjoyment, subjective norm, perceived ease of use, and perceived usefulness, were evaluated as predictors for the intention to use 3D Organon for learning. GETAMEL showed that students highly perceived 3D Organon for learning anatomy across all variables. Students generally felt confident using 3D Organon, enjoyed their experience with the software, found it useful and easy to use, and expressed their intention to use it to study other anatomical topics.

Interestingly, enjoyment was the highest‐rated aspect of 3D Organon, suggesting that this is the software's most prominent feature. Acceptance of 3D Organon was consistent across cohorts, indicating that its acceptance did not depend on students' discipline. In addition, overall self‐perceived eye and ear anatomy comprehension significantly improved after the learning session. This study provides evidence for utilizing 3D Organon as an additional anatomy learning resource.

### 3D Organon perception and influencing factors

Study findings affirmed most of the relationships proposed in the GETAMEL.^52^ Additionally, the relationships between GETAMEL's external variables and the TAM's main variables were observed in the study, lending support to GETAMEL as a robust technology acceptance measure.

Results showed that self‐efficacy, enjoyment, and computer anxiety significantly predicted the ease of use, contributing to 91% of its variance. The significant positive relationship found in this study between self‐efficacy and perceived ease of use is consistent with previous e‐learning research across various disciplines.^43^, ^51^, ^55^, ^59^, ^62^, ^71^, ^93^, ^94^, ^95^ Similarly, the significant positive relationship between enjoyment and perceived ease of use aligns with the e‐learning literature.^43^, ^51^, ^55^, ^71^, ^96^ The negative association between computer anxiety and perceived ease of use observed in the study is also supported by prior work.^45^, ^59^, ^71^, ^95^ Findings indicate that students who were confident about using 3D Organon used it comfortably, enjoyed using it for learning, and perceived it as easy to use. Further analysis showed that enjoyment was the strongest predictor of perceived ease of use (*β* = 0.78), and these findings partially agree with prior GETAMEL's findings where enjoyment was found as the second strongest predictor of perceived ease of use after self‐efficacy.^55^

In line with the GETAMEL,^52^ perceived usefulness was significantly predicted by self‐efficacy and enjoyment, which together accounted for 83% of its variance. The impact of self‐efficacy on perceived usefulness found in the study is supported by previous work,^62^, ^93^, ^94^ and the effect of enjoyment on perceived usefulness is also consistent with the e‐learning literature.^43^, ^51^, ^55^, ^96^ These findings indicate that if students felt confident about using 3D Organon to learn anatomy and enjoyed using it, they would also view it as useful. The influence of perceived ease of use on perceived usefulness, as proposed by the modified TAM,^48^ was also observed in this study. This finding is consistent with prior research in e‐learning^43^, ^45^, ^55^, ^59^, ^62^, ^71^, ^93^, ^96^, ^97^, ^98^ and mobile‐assisted anatomy learning.^99^ Our findings echoed Abdullah et al.^55^ in finding the perceived ease of use as the strongest predictor of perceived usefulness (*β* = 1.02).^55^

The finding that students' confidence and comfort while using 3D Organon were associated with their perceptions of 3D Organon is noteworthy from several perspectives. Due to the novelty of VR technology and the lack of VR experience among students, one would expect self‐efficacy and anxiety to be affected; however, the results show high levels of confidence and comfort while using 3D Organon. This observation could be because these students already possess the necessary skills to deal with such technology. Furthermore, students in the current study reported higher anatomy understanding following the learning session, indicating that they used 3D Organon effectively during the learning session. In discussing the effect of computer self‐efficacy on academic achievement, low computer self‐efficacy and lack of computer skills have been reported by others to be associated with ineffective computer‐based learning,^100^ shedding light on the critical role of computer skills and self‐efficacy in technology‐based learning. Equally important is the finding that enjoyment was associated with positive perceptions toward 3D Organon. From an academic standpoint, inherently enjoyable learning tasks can promote an intrinsic motivation to learn,^100^, ^101^ enhance engagement, and perhaps even contribute to academic success.^102^ A previous study found an association between enjoyment, the use of computers, and academic achievement,^100^ lending evidence to the role of enjoyment in computer‐based learning. In light of previous research on these factors, our findings suggest that self‐efficacy, anxiety, and enjoyment are essential indicators of the successful integration of VR anatomy software into anatomy education.

In contrast to prior studies,^43^, ^45^, ^51^, ^52^, ^55^, ^71^, ^93^, ^95^ the current study found no evidence of subjective norm influencing perceived ease of use or perceived usefulness. This suggests that students' perceptions of 3D Organon were not influenced by others. Specifically, only half the students had prior VR experience, and the mean subjective norm score was on the margin of agreement, close to neutral (*μ* = 3.53). This observation implies that students were uncertain about the impact of others' opinions on their perceptions toward the software. Given the limited integration of VR software in anatomy education, students' viewpoints about 3D Organon may not be well‐established. While this finding contradicts the GETAMEL, it can be viewed positively from an educational perspective. The positive perception of using 3D Organon for learning does not depend on other's opinions but rather on factors related to the learners. It is argued that the educational setting and the technology employed for e‐learning can contribute to the discrepancy in subjective norm findings among e‐learning studies.^103^ Hence, additional research is necessary to demonstrate the precise influence of subjective norm on student acceptance and usage of e‐learning.

Prior research has consistently identified perceived usefulness as a stronger predictor of behavioral intention to use technology.^53^, ^55^ In anatomy education, a study that evaluated a mobile anatomy application (4natomy) using the TAM found that perceived usefulness was the best predictor of students' intention to use the app to learn anatomy.^99^ However, in the present study, the behavioral intention to use 3D Organon was more influenced by perceived ease of use (*β* = 1.01) than perceived usefulness (*β* = 0.87). Together, the perceived ease of use and perceived usefulness explained 86% of the variance in the behavioral intention to use 3D Organon for learning. These findings support the TAM's central concept that the behavioral intention to use a technology relies on how usable and valuable it seems to its users.^48^, ^99^

In summary, VR anatomy software that offers students comfort, confidence, ease, and enjoyment is more likely to be perceived as valuable and user‐friendly, resulting in greater acceptance in anatomy education. Considering these factors when selecting VR anatomy software, emphasizing enjoyment and usability, can enhance student acceptance, adoption, and learning outcomes.

### Practical implications

Among the predictors of perceived ease of use, enjoyment was the most dominant determinant of students' perception of 3D Organon, exhibiting the highest *β* weight (*β* = 0.78). Thus, it should be particularly emphasized when integrating 3D Organon and similar software into anatomy education. This can be ensured by designing engaging 3D Organon‐based learning activities, including game‐like learning tasks, or using such software for group‐based learning. Before implementation in the classroom, enjoyment should be addressed in the developing phase of VR anatomy software to design software capable of capturing students' attention and guaranteeing acceptance.

Perceived ease of use had the strongest impact on both perceived usefulness (*β* = 1.02) and behavioral intention to use 3D Organon (*β* = 1.01). Incorporating user‐friendly VR anatomy software can significantly enhance the acceptance and adoption of VR learning systems for anatomy education. The choice of VR device and anatomy software should be based on their usability, among other criteria. Promoting familiarity with the VR system before the learning tasks would enhance usability and improve students' confidence (self‐efficacy) in handling the system. This can be tackled by arranging on‐campus practice sessions where students practice using the system, as described in this study, and by providing the necessary technical support within the classroom. Students' responses to the open‐ended questions highlighted challenges such as technical difficulties with 3D Organon and the VR device, underscoring the need for robust technical support to enhance the effectiveness of VR for learning. Additionally, students recommended extended VR training and more frequent learning sessions to maximize learning outcomes.

### Anatomy perception

Overall, self‐perceived anatomy comprehension significantly improved after the VR learning session; however, the cohort‐based analysis showed only a significant improvement in ear anatomy comprehension among the MSc students. The MSc students reported a mean of 2.11 for pre‐VR ear comprehension, compared with 2.35 in pre‐VR eye comprehension, suggesting that ear anatomy is more complex to this cohort. By contrast, the neuroscience cohort reported a mean of 2.51 in pre‐VR ear comprehension, indicating high initial understanding that could have masked significant improvements. Considering that neuroscience students studied neuroanatomy, which demands high spatial comprehension,^104^ this could have positively influenced their overall understanding of 3D anatomy. Despite this, higher post‐VR anatomy comprehension among both cohorts suggests that students believed 3D Organon assisted their comprehension of both structures, supporting its use as a 3D visualization tool in learning spatially complex structures.

### Limitations

Data collected via the GETAMEL and self‐perceived anatomy comprehension questions were self‐reported, making them inherently subjective. Additionally, anatomy comprehension was not objectively measured using standardized tests, which could have provided a more accurate assessment of learning outcomes. However, this study focused primarily on the impact of VR‐based anatomy learning from the student's perspective. The small sample size further limits the generalizability of the findings. However, including students from two disciplines enhances the robustness of the results. Additionally, the novelty of VR may have introduced a bias toward 3D Organon, and the potential role of prior experience with the software was not explored.

## CONCLUSION

The use of VR visualization technologies holds great promise in anatomy education and is supported by empirical evidence. However, the effect of VR anatomy learning can only be fully unlocked if students are willing to use it. This study aimed to objectively measure the extent to which the students accept using 3D Organon as an anatomy learning tool based on the GETAMEL and TAM perspectives. Additionally, this study examined the influence of various factors on the level of 3D Organon acceptance for learning anatomy. The findings of this study could be of great value to education providers interested in technology‐enhanced learning in anatomy and medical education, along with designers and developers of 3D anatomy e‐learning systems. Students perceived 3D Organon positively and contributed to a better understanding of two complex anatomical structures. Students felt confident, comfortable, and engaged while using 3D Organon, likely contributing to their positive perceptions of the software as an anatomy learning tool. Due to its ease of use and helpfulness, students expressed their intentions to continue using 3D Organon for further anatomy learning. Among the factors, enjoyment and ease of use exhibit the most significant effect on students' acceptance of 3D Organon; therefore, they must be prioritized in developing 3D Organon‐based learning activities. Although this study addressed the five most used factors influencing technology acceptance, future research can explore other factors to understand the acceptance of VR technology in the context of anatomy learning and expand the study to include larger student groups.

## AUTHOR CONTRIBUTIONS

**Sarah Alturkustani:** Data curation; formal analysis; investigation; methodology; writing – original draft. **Ashley Durfee:** Conceptualization; data curation; formal analysis; investigation; methodology; writing – original draft; writing – review and editing. **Olivia F. O'Leary:** Conceptualization; investigation; methodology; writing – review and editing. **Siobhain M. O'Mahony:** Conceptualization; methodology; supervision; writing – review and editing. **Conor O'Mahony:** Conceptualization; investigation; methodology; writing – review and editing. **Mutahira Lone:** Conceptualization; investigation; methodology; resources; supervision; writing – review and editing. **Andreea Factor:** Conceptualization; investigation; methodology; resources; supervision; writing – review and editing.

## ETHICS STATEMENT

Ethical approval was obtained from the Social Research Ethics Committee (SREC) at University College Cork (Log 2023‐076).

## Supporting information


Data S1.

Data S2.
