# Supplementary material for: Measuring students' perceptions of virtual reality for learning anatomy using the general extended technology acceptance model for E‐learning
Source: Anat Sci Educ. 2025 May 23;18(6):579–95. doi: 10.1002/ase.70045 (PMC12135465; doi:10.1002/ase.70045)
Supplement: Supplementary file 1 — Data S1. [file ASE-18-579-s001.docx]

**Students’ perception of the use of Virtual Reality for anatomy learning**

We would be very grateful if you could complete this questionnaire, which can take up to 10 minutes. The questionnaire is composed of 2 sections addressing your background, perception of using virtual reality to learn anatomy, and comprehension of eye and ear anatomy. This questionnaire is part of an MSc thesis/PhD thesis. YOUR ANSWERS ARE VERY IMPORTANT TO US and will help us achieve our goals. Participation is entirely voluntary. All responses will be treated as strictly confidential and will be used for research and academic purposes only.

If you have any questions regarding this questionnaire, please contact

*Sarah Alturkustani*

PhD student

*Department of Anatomy and Neuroscience*

Email: [119226571@umail.ucc.ie](mailto:119226571@umail.ucc.ie)

I have read the provided information and agree to participate in this research.

I am above the age of 18.

**Section 1 (perception of VR)**

1. Please write the last five digits of your phone number

|  |
| --- |

1. For each of the next statements, please tick the response that best characterises how you feel about the statement, where:

1 = Strongly agree, 2 = Moderately agree, 3 = Slightly agree, 4 = Neutral, and 5 = Slightly disagree, 6 = Moderately disagree, 7 = Strongly disagree

| 1. **Self-efficacy** | | **1** | **2** | **3** | **4** | **5** | **6** | **7** |
| --- | --- | --- | --- | --- | --- | --- | --- | --- |
| 1.1 | I am confident to use the VR anatomy software if there is no one around to show me how to do it |  |  |  |  |  |  |  |
| 1.2 | I am confident to use the VR anatomy software even if I have never used such a system before |  |  |  |  |  |  |  |
| 1.3 | I am confident to use the VR anatomy software even if I have only the software manual for reference |  |  |  |  |  |  |  |
| 1. **VR anatomy software anxiety** | | **1** | **2** | **3** | **4** | **5** | **6** | **7** |
| 2.1 | Working with VR anatomy software does not make me nervous |  |  |  |  |  |  |  |
| 2.2 | VR anatomy software makes me uncomfortable |  |  |  |  |  |  |  |
| 2.3 | VR anatomy software makes me uneasy |  |  |  |  |  |  |  |
| 1. **Enjoyment** | | **1** | **2** | **3** | **4** | **5** | **6** | **7** |
| 3.1 | I find using the VR anatomy software enjoyable |  |  |  |  |  |  |  |
| 3.2 | The actual process of using the VR anatomy software is pleasant |  |  |  |  |  |  |  |
| 1. **Subjective norm** | | **1** | **2** | **3** | **4** | **5** | **6** | **7** |
| 4.1 | People who influence my behaviour would think that I should use the VR anatomy software |  |  |  |  |  |  |  |
| 4.2 | I am influenced by my peers and my lecturers in using the VR anatomy software |  |  |  |  |  |  |  |
| 1. **Perceive ease of use** | | **1** | **2** | **3** | **4** | **5** | **6** | **7** |
| 5.1 | I find it easy to use the VR headsets to navigate through the anatomy software |  |  |  |  |  |  |  |
| 5.2 | My interaction with the VR anatomy software is clear and understandable |  |  |  |  |  |  |  |
| 5.3 | I would find the VR anatomy software to be flexible to interact with |  |  |  |  |  |  |  |
| 5.4 | It would be easy for me to become skilful at using the VR anatomy software |  |  |  |  |  |  |  |
| 5.5 | I would find the VR anatomy software helpful in visualizing the structures |  |  |  |  |  |  |  |
| 1. **Perceived usefulness** | | **1** | **2** | **3** | **4** | **5** | **6** | **7** |
| 6.1 | Using the VR anatomy software allows me to accomplish learning tasks more quickly |  |  |  |  |  |  |  |
| 6.2 | Using the VR anatomy software improves my learning performance |  |  |  |  |  |  |  |
| 6.3 | Using the VR anatomy software enhances my effectiveness in learning |  |  |  |  |  |  |  |
| 6.4 | Using the VR anatomy software would make learning easier |  |  |  |  |  |  |  |
| 1. **Behavioural intention to use the VR anatomy software** | | **1** | **2** | **3** | **4** | **5** | **6** | **7** |
| 7.1 | Assuming I had access to the VR anatomy software, I would use it for learning other anatomical topics |  |  |  |  |  |  |  |
| 7.2 | Given that I had access to the VR anatomy software, I predict that I would use it for learning other anatomical topics |  |  |  |  |  |  |  |
| 7.3 | I plan to use the VR anatomy software in the future |  |  |  |  |  |  |  |

1. Did you experience any physical discomfort while using the VR anatomy software?

Yes

No

1. If yes, please describe the discomfort.

|  |
| --- |

1. Which aspect of the VR anatomy software did you like the most and why?

|  |
| --- |

1. Which aspect of the VR anatomy software did you like the least and why?

|  |
| --- |

1. What anatomical topic/structure do you think the VR would be an effective learning tool and why?

|  |
| --- |

1. How does VR compare to conventional methods in learning the eye and ear anatomy? (i.e. lectures, plastic models, textbooks).

|  |
| --- |

1. Using VR, do you feel more motivated to learn anatomy?

|  |
| --- |

1. How can we improve your anatomy learning experience using VR?

|  |
| --- |

**Section 2 (anatomy comprehension)**

1. After using the VR, please rate your comprehension of the anatomy of the following structures using the scale provided.

1 = Not understood, 2 = Partially understood, 3 = Well understood

| 1. **Eye** | | **1** | **2** | **3** |
| --- | --- | --- | --- | --- |
| 1.1 | Extraocular muscles |  |  |  |
| 1.2 | The fibrous layer of the eyeball |  |  |  |
| 1.3 | The vascular layer of the eyeball |  |  |  |
| 1.4 | The nervous layer of the eyeball |  |  |  |
| 1.5 | The lens |  |  |  |
| 1.6 | The vitreous body |  |  |  |
| 1. **Ear** | | **1** | **2** | **3** |
| 2.1 | Tympanic membrane |  |  |  |
| 2.2 | Tympanic cavity (middle ear) |  |  |  |
| 2.3 | Auditory ossicles |  |  |  |
| 2.4 | Inner ear |  |  |  |
| 2.5 | Cochlea |  |  |  |
| 2.6 | Vestibule |  |  |  |
| 2.7 | Semi-circular canals |  |  |  |

**Thank you for completing this questionnaire!**
