# Supplementary material for: Measuring students' perceptions of virtual reality for learning anatomy using the general extended technology acceptance model for E‐learning
Source: Anat Sci Educ. 2025 May 23;18(6):579–95. doi: 10.1002/ase.70045 (PMC12135465; doi:10.1002/ase.70045)
Supplement: Supplementary file 2 — Data S2. [file ASE-18-579-s002.docx]

**Students’ perception of the use of Virtual Reality for anatomy learning**

We would be very grateful if you could complete this questionnaire, which can take up to 10 minutes. The questionnaire is composed of 2 sections addressing your background and comprehension of eye and ear anatomy. This questionnaire is part of an MSc thesis/PhD thesis. YOUR ANSWERS ARE VERY IMPORTANT TO US and will help us achieve our goals. Participation is entirely voluntary. All responses will be treated as strictly confidential and will be used for research and academic purposes only.

If you have any questions regarding this questionnaire, please contact

*Sarah Alturkustani*

PhD student

*Department of Anatomy and Neuroscience*

Email: [119226571@umail.ucc.ie](mailto:119226571@umail.ucc.ie)

I have read the provided information and agree to participate in this research.

I am above the age of 18.

**Section 1 (background)**

**Please tell us about yourself by answering the following questions**

1. Please write the last five digits of your phone number

|  |
| --- |

1. Please state your age

|  |
| --- |

1. Please state your country of origin

|  |
| --- |

1. How would you describe your gender?

Male

Female

I prefer to self-describe as____________________________

I prefer not to say

1. Please state the highest level of school you have completed

Secondary level qualification or equivalent

Attended college but did not receive degree

Bachelor’s degree

Master’s degree

PhD degree

Other. Please specify______________________

1. Were you previously enrolled in any anatomy course?

Yes

No

1. If yes, briefly describe the course

|  |
| --- |

1. Have you learnt the anatomy of the eye before?

Yes

No

1. Have you learnt the anatomy of the ear before?

Yes

No

Have you used any digital 3D anatomy atlas before? Such as computer-based anatomy models, mobile applications, etc.

Yes

No

1. How often do you use digital 3D anatomy atlases?

Very frequently

Frequently

Occasionally

Rarely

Never

1. Do you have any experience with video games?

Yes

No

1. If yes, please specify the length of your video game experience.

|  |
| --- |

1. Do you have any experience with Virtual Reality? (Virtual reality is simulated experience using head-mounted display that fully immerses the user's senses in a 3D environment that mimics real-world properties)

Yes

No

1. If yes, please specify the length of your VR experience.

|  |
| --- |

1. Where have you utilised VR in the past?

Video games

At a museum

At an educational conference

In a classroom

Other. Please specify____________________________

Not applicable

1. How would you describe your experience with VR?

Positive

Neutral

Negative

Not applicable

1. On a scale from 1-10 (with 1 being the lowest, and 10 the highest), how would you rate your VR experience?

| 1 | 2 | 3 | 4 | 5 | 6 | 7 | 8 | 9 | 10 |
| --- | --- | --- | --- | --- | --- | --- | --- | --- | --- |

1. Would you be interested in using VR to learn anatomy?

Yes

No

Unsure

1. Have you been diagnosed with any of the following?
2. Learning disabilities  Yes. Please specify__________________  No
3. Vision problems  Yes. Please specify__________________  No
4. Loss of balance/equilibrium  Yes. Please specify__________________  No
5. If you previously used a VR headset, did you experience any physical discomfort while using it? Please choose all that apply.

Headache

Nausea

Double vision

Blurred vision

Dizziness

Motion sickness

Loss of balance

Other. Please specify____________________________

Not applicable

**Section 2 (anatomy comprehension)**

1. Please rate your comprehension of the anatomy of the following structures using the scale provided.

1 = Not understood, 2 = Partially understood, 3 = Well understood

| 1. **Eye** | | **1** | **2** | **3** |
| --- | --- | --- | --- | --- |
| 1.1 | Extraocular muscles |  |  |  |
| 1.2 | The fibrous layer of the eyeball |  |  |  |
| 1.3 | The vascular layer of the eyeball |  |  |  |
| 1.4 | The nervous layer of the eyeball |  |  |  |
| 1.5 | The lens |  |  |  |
| 1.6 | The vitreous body |  |  |  |
| 1. **Ear** | | **1** | **2** | **3** |
| 2.1 | Tympanic membrane |  |  |  |
| 2.2 | Tympanic cavity (middle ear) |  |  |  |
| 2.3 | Auditory ossicles |  |  |  |
| 2.4 | Inner ear |  |  |  |
| 2.5 | Cochlea |  |  |  |
| 2.6 | Vestibule |  |  |  |
| 2.7 | Semi-circular canals |  |  |  |

**Thank you for completing this questionnaire!**
